# Unequal access to liquid biopsy in colorectal cancer care: a cross-sectional survey of clinicians across hospital tiers in Zhejiang, China

**DOI:** 10.3389/fcell.2026.1870567

**Published:** 2026-08-06

**Authors:** Sheng Li, Zhonglei Shen, Youchang Sun, Yitong Hu, Yisheng Cao

**Affiliations:** 1 Department of Anorectal Surgery, Ningbo No. 2 Hospital, Wenzhou Medical University, Ningbo, Zhejiang, China; 2 Health Science Center, Ningbo University, Ningbo, Zhejiang, China

**Keywords:** accessibility, circulating tumor DNA, clinical adoption, colorectal cancer, liquid biopsy

## Abstract

**Objective:**

Liquid biopsy is increasingly used in colorectal cancer (CRC) management, but its real-world adoption remains limited by cost, evidence gaps, and uneven institutional capacity. This study assessed the clinical use, accessibility, and barriers to liquid biopsy among CRC-related clinicians in Zhejiang Province, China.

**Methods:**

A cross-sectional online survey was conducted among clinicians involved in CRC diagnosis and treatment. The questionnaire collected data on respondent characteristics, liquid biopsy use, clinical application scenarios, perceived barriers, and willingness for future adoption. Descriptive statistics, univariate logistic regression, and multivariable logistic regression were used for analysis.

**Results:**

Among 246 valid respondents, more than half reported having used liquid biopsy in CRC practice. Circulating tumor DNA was the most frequently used modality. The main applications were anti-EGFR resistance monitoring, postoperative minimal residual disease assessment, and recurrence risk monitoring. The leading barrier was high testing cost and lack of insurance coverage, followed by insufficient clinical evidence and uncertainty in result interpretation. Liquid biopsy accessibility was higher among clinicians from Grade III Class A hospitals, dedicated CRC treatment centers, and institutions treating more than 100 CRC cases annually; multivariable analysis identified annual case volume >100 cases as the sole independent predictor. A total of 89.8% of respondents expressed willingness to incorporate liquid biopsy into routine practice if reimbursement and accessibility improved.

**Conclusion:**

Liquid biopsy shows growing clinical acceptance in CRC care, but its adoption remains constrained by economic, evidentiary, and institutional barriers. Reimbursement support, standardized pathways, and targeted education are needed to promote equitable implementation.

## 1 Introduction

Colorectal cancer (CRC) is the second leading cause of cancer and cancer-related mortality worldwide (Siegel et al., 2024). Its incidence and mortality rates have been rising annually, posing a serious threat to public health and constituting a significant public health challenge. Diagnosis, prognosis, and treatment monitoring of CRC largely depend on tissue biopsy. However, a major issue is that repeated biopsies for disease progression monitoring are challenging due to repeated tissue trauma and poor patient compliance. Furthermore, a single biopsy typically fails to capture patient heterogeneity, cannot reflect the evolving full spectrum of cancer gene expression, and is constrained by limitations such as tissue sampling location, poor sensitivity and accuracy, and high procedural costs (Hamzehzadeh et al., 2017; Baxter and Rabeneck, 2009). Compared to tissue biopsy, liquid biopsy (LB) has gained widespread adoption in oncology as a non-invasive, repeatable molecular detection method (Marrugo-Ramírez et al., 2018). It dynamically captures tumor molecular characteristics and evolutionary processes through circulating tumor DNA (ctDNA), circulating tumor cells (CTCs), exosomes, and methylation markers in bodily fluids like blood (Batool et al., 2023). Compared to traditional tissue biopsy, LB offers advantages in non-invasiveness, effective monitoring, and longitudinal assessment of treatment dynamics, particularly in scenarios where tissue sample acquisition is challenging. In recent years, with advances in detection technologies such as high-sensitivity NGS platforms, LB applications are transitioning from exploratory research to clinical practice.

In CRC, LB’s potential applications are particularly prominent: Postoperative detection of minimal residual disease (MRD) aids in recurrence risk stratification and adjuvant therapy decisions (Torresan et al., 2024); ctDNA monitoring during follow-up can signal recurrence earlier than imaging (Schøler et al., 2017; Wang et al., 2019); for translational therapy and oligometastatic disease management, LB serves as a critical reference for efficacy assessment and surgical timing determination (Mauri et al., 2022); For metastatic CRC patients, LB can track resistance mutations in RAS, BRAF, and EGFR pathways (Torresan et al., 2024; Mauri et al., 2022; van 't Erve et al., 2020), informing re-challenge strategies or targeted therapy selection. As detection technologies advance and research evidence accumulates, LB’s role in CRC clinical management is progressively transitioning from exploration to practice. However, the path from cutting-edge research potential to widespread clinical adoption remains fraught with challenges.

Despite promising prospects, a distinct “evidence-practice gap” persists between the application of LB in real-world clinical settings and its substantial potential demonstrated in research (Mauri et al., 2022). Key barriers include relatively high testing costs, incomplete healthcare reimbursement policies, uneven distribution of testing platforms and laboratory accreditation, and insufficient clinician confidence in interpreting results and recognizing their evidence-based value (Ignatiadis et al., 2021; Febbo et al., 2024; Heidrich et al., 2021; Norman et al., 2018). Furthermore, existing research predominantly focuses on technical feasibility and prognostic prediction, lacking large-scale, multicenter evidence (Cescon et al., 2020; Veronese et al., 2025) to clarify “which populations benefit most, when testing is most appropriate, and how results influence treatment decisions.” These practical challenges prevent LB from being fully integrated into routine clinical care for CRC. Particularly in China, despite rapid advances in medical technology in recent years, systematic data on the current status, regional disparities, and barriers to adoption of LB in clinical practice remain scarce, limiting the formulation of relevant health policies. Therefore, there is an urgent need to investigate the current usage, accessibility, and primary barriers among frontline clinicians to identify bottlenecks and priority issues for resolution, thereby providing evidence for establishing standardized pathways and policy support at the regional level.

Against this backdrop, we designed and conducted a cross-sectional survey study across Zhejiang Province, China, aiming to systematically evaluate the usage, accessibility, primary barriers, and future development directions of LB in CRC clinical practice. This study targeted professionals in colorectal surgery, medical oncology, radiation oncology, pathology, and related disciplines, covering hospitals and treatment centers at various levels. Through a standardized online questionnaire, we sought to map the true landscape of LB application for CRC in Zhejiang Province and identify key factors influencing its clinical adoption. We hope this study will inform subsequent multicenter prospective research, promote standardized application of LB in CRC diagnosis and treatment, and provide a reference basis for medical insurance reimbursement and policy formulation.

## 2 Materials and methods

This study employed a cross-sectional survey design. The research subjects comprised medical personnel engaged in colorectal cancer diagnosis, treatment, or related work within Zhejiang Province, including physicians and researchers from colorectal surgery, medical oncology, radiation oncology, pathology, and other relevant disciplines. Interns and senior residents who had participated in CRC diagnosis or treatment within the past 12 months were also eligible for inclusion. We widely distributed online questionnaires through provincial-level academic societies, specialty alliances, and academic conferences to ensure representation across different regions and levels of medical institutions. The questionnaire was designed by the research team based on existing literature and expert discussions, covering three core modules: ① Demographic and professional background of respondents; ② Accessibility and usage of LB in CRC; ③ Clinical application scenarios, primary barriers, and future application intentions. Prior to formal implementation, the questionnaire was reviewed for face validity by three senior clinicians with expertise in CRC management (representing colorectal surgery, medical oncology, and pathology), who assessed item clarity, clinical relevance, and comprehensiveness of response options. A pilot test was subsequently conducted among five clinicians meeting the eligibility criteria who did not participate in the main survey; items with ambiguous wording or low response completion rates were revised accordingly. All questionnaire items were treated as independent measures of distinct constructs, including LB accessibility, clinical application scenarios, perceived barriers, and willingness for future adoption. Cronbach’s alpha is therefore not applicable to the instrument as a whole, as no multi-item scale was constructed. The final version of the questionnaire is available in Supplementary Material S1. All respondents participated after providing online informed consent, with anonymous completion to safeguard data privacy and authenticity. System restrictions prevented duplicate submissions, and questionnaires lacking complete responses to key sections (LB application and barriers) were excluded from analysis.

### 2.1 Sample size calculation

Epidemiological research formula was used to calculate sample size:

$$n=\frac{z^{2}p\left(1-p\right)}{e^{2}}$$

Assuming a conservative proportion of 0.5 and a margin of error of 0.05, the required sample size at a 95% confidence level (Z = 1.96) was calculated to be 385 participants.

Due to feasibility constraints and the exploratory nature of this regional survey, a total of 246 valid responses were ultimately included. With N = 246 and an assumed proportion of 0.5, the achieved margin of error can be calculated as: $e=Z\sqrt{\frac{p\left(1-p\right)}{n}}$ = 1.96 $\sqrt{\frac{0.5\times \left(1-0.5\right)}{246}}$ ​≈0.0625. This sample size shortfall increases the margin of error from the intended ±5% to approximately ±6.3% at the 95% confidence level, and may reduce the precision of odds ratio estimates

### 2.2 Data collection process

Data collection was performed from September to November 2025, utilized the Wenjuanxing platform (https://www.wjx.cn/), a widely adopted professional survey tool in China supporting robust data management and quality control. Data anonymization occurred during entry, with no personally identifiable information collected. To ensure data validity and minimize duplicate submissions, responses were limited to one per unique IP address. Participants accessed the survey via quick response code scanning and completed the questionnaire anonymously on mobile devices or computers to encourage honest and impartial responses. The platform ensures secure data storage, audit trails, and role-based access permissions. Weekly monitoring tracked regional and specialty distribution. Quality checks included duplicate data removal, completeness validation, and feedback logic consistency verification.

### 2.3 Research variables and outcome measures

The primary outcome of this study was the utilization rate of LB in colorectal cancer diagnosis and treatment among Zhejiang Province clinicians, along with its primary barriers.

#### 2.3.1 Primary outcome measures

Whether LB has been applied in CRC patients (Yes/No); Primary barriers to LB adoption identified by respondents (including high cost/lack of reimbursement, insufficient clinical evidence, limited accessibility, difficulty interpreting results, inadequate laboratory quality control, poor patient acceptance, etc.).

#### 2.3.2 Secondary outcomes

Accessibility of LB: Whether the respondent’s hospital possesses in-house testing capabilities, whether external testing is required, types of tests (ctDNA, CTCs, methylation, fragmentomics, etc.), and turnaround time for reports; Usage scenarios: Postoperative MRD monitoring, recurrence follow-up, response assessment to translational therapies, monitoring of anti-EGFR resistance and re-challenge, prognosis evaluation, clinical research, etc.,; Attitude toward future adoption: Willingness to incorporate into routine practice if testing becomes more accessible or covered by insurance; Respondent demographics: Age group, specialty category, professional title, hospital tier, presence of CRC center, annual CRC case volume.

#### 2.3.3 Stratification and subgroup analysis variables

Stratified by hospital tier (Grade III Class A hospital, Grade III hospital and Grade II hospital and below), CRC center status, specialty category (surgery/internal medicine/radiation oncology/pathology, etc.), and annual case volume (high/low) to compare differences in LB utilization rates, barriers, and application scenarios across groups.

All data were collected via the Wenjuanxing platform, double-checked, and imported into statistical software (SPSS 26.0) for analysis.

### 2.4 Statistical analysis

Descriptive statistics summarized respondent characteristics and survey responses. Categorical variables were presented as frequencies and percentages. Intergroup comparisons employed chi-square tests or Fisher’s exact tests (for categorical variables) and Mann-Whitney U tests or Kruskal–Wallis tests (for ordinal variables). Univariate logistic regression was first performed to examine the association between each candidate variable and LB accessibility individually. Prior to multivariable modeling, variance inflation factors (VIFs) were calculated to assess multicollinearity among institutional predictors (hospital tier, CRC center designation, and annual case volume). Hospital tier was excluded from the multivariable model due to a VIF exceeding 10 (VIF = 11.2), indicative of substantial collinearity with annual case volume. The final multivariable logistic regression model included CRC center designation, annual case volume, specialty category, and professional title, with results presented as adjusted odds ratios (aOR) and 95% confidence intervals (CI). Model fit was evaluated using the Hosmer-Lemeshow goodness-of-fit test. Missing data in non-critical variables were handled using the full case analysis method, and sensitivity analyses were conducted to assess the robustness of the results. Visualizations included Sankey diagrams to illustrate adoption pathways and bar charts to present barriers and application scenarios.

All analyses were performed using SPSS software. A two-tailed P value <0.05 was considered statistically significant.

## 3 Results

**FIGURE 1:**
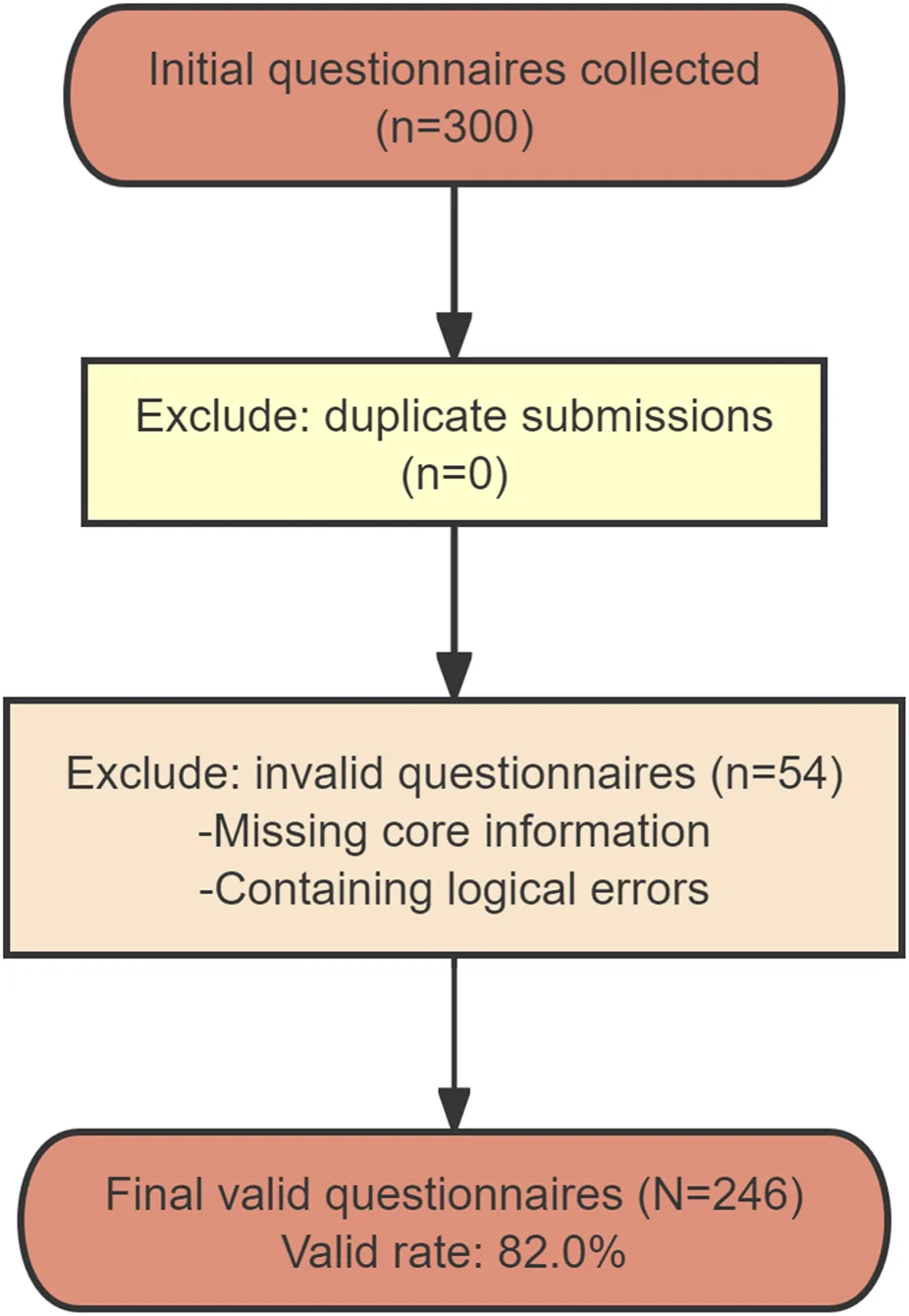
Study Subject Screening Flowchart. A total of 300 questionnaires were initially collected. No duplicate submissions were identified. After excluding 54 questionnaires due to missing core information or logical inconsistencies, 246 valid questionnaires were included in the final analysis.

During the study period, we leveraged the Zhejiang Province Multidisciplinary Collaboration Network for Colorectal Cancer to collect questionnaires through a combination of online and offline methods. The subject selection process is detailed in Figure 1. A total of 300 questionnaires were returned. After excluding 54 invalid responses, 246 valid questionnaires were included, yielding a valid questionnaire rate of 82.0% among returned questionnaires. After submission, the system automatically detected duplicates to ensure each physician submitted only once. All data underwent manual verification before final analysis.

### 3.1 Baseline characteristics of respondents

**FIGURE 2:**
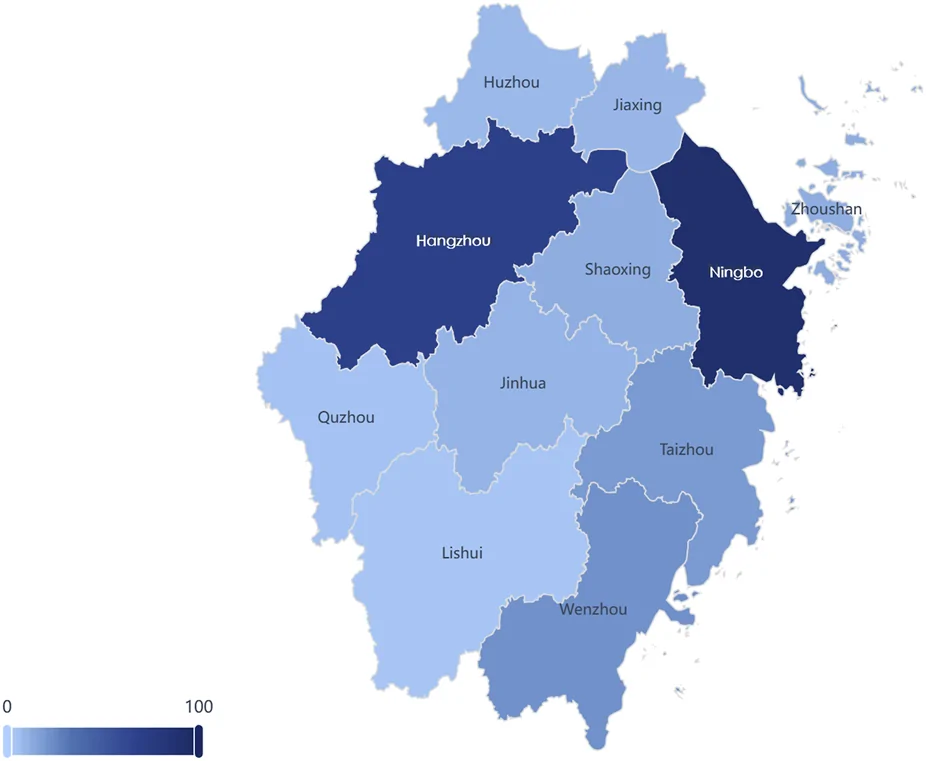
Geographic Distribution of Study Participants (N = 246). The map illustrates the regional distribution of respondents by city. Color intensity represents the number of participants from each region, with darker shades indicating higher participation. Hangzhou and Ningbo contributed the largest number of respondents.

The 246 respondents included in the final analysis represented the entire province of Zhejiang, with their geographic distribution shown in Figure 2. Respondents were predominantly male (76.8%), middle-aged and young adults aged 31–50 (81.3%), and held the title of attending physician or higher (91.1%). The vast majority of respondents worked at Grade III Class A hospitals (68.3%) where colorectal cancer diagnosis and treatment centers were established (58.5%). Centers treating over 100 CRC cases annually accounted for 60.2%. By specialty, colorectal surgeons (42.7%) and medical oncologists (35.0%) were the primary participants. Detailed baseline characteristics are presented in Table 1.

**TABLE 1:** Baseline characteristics of respondents (N = 246).

Characteristic	Category	Frequency (n)	Percentage (%)
Gender	Male	189	76.8%
	Female	57	23.2%
Age group	≤30 years old	25	10.2%
	31–40 years old	112	45.5%
	41–50 years old	88	35.8%
	>50 years old	21	8.5%
Professional title	Resident/Intern physician	22	8.9%
	Attending physician	120	48.8%
	Associate Chief physician	70	28.5%
	Chief physician	34	13.8%
Hospital level	Grade III Class A	168	68.3%
	Grade III (non-Class A)	45	18.3%
	Grade II and below	33	13.4%
Discipline category	Colorectal surgery	105	42.7%
	Medical oncology	86	35.0%
	Radiation oncology	24	9.8%
	Pathology/Other	31	12.6%
Designated CRC treatment center	Yes	144	58.5%
	No	102	41.5%
Annual CRC case volume	<50 cases	48	19.5%
	50–100 cases	50	20.3%
	>100 cases	148	60.2%

### 3.2 Accessibility and utilization of LB

**FIGURE 3:**
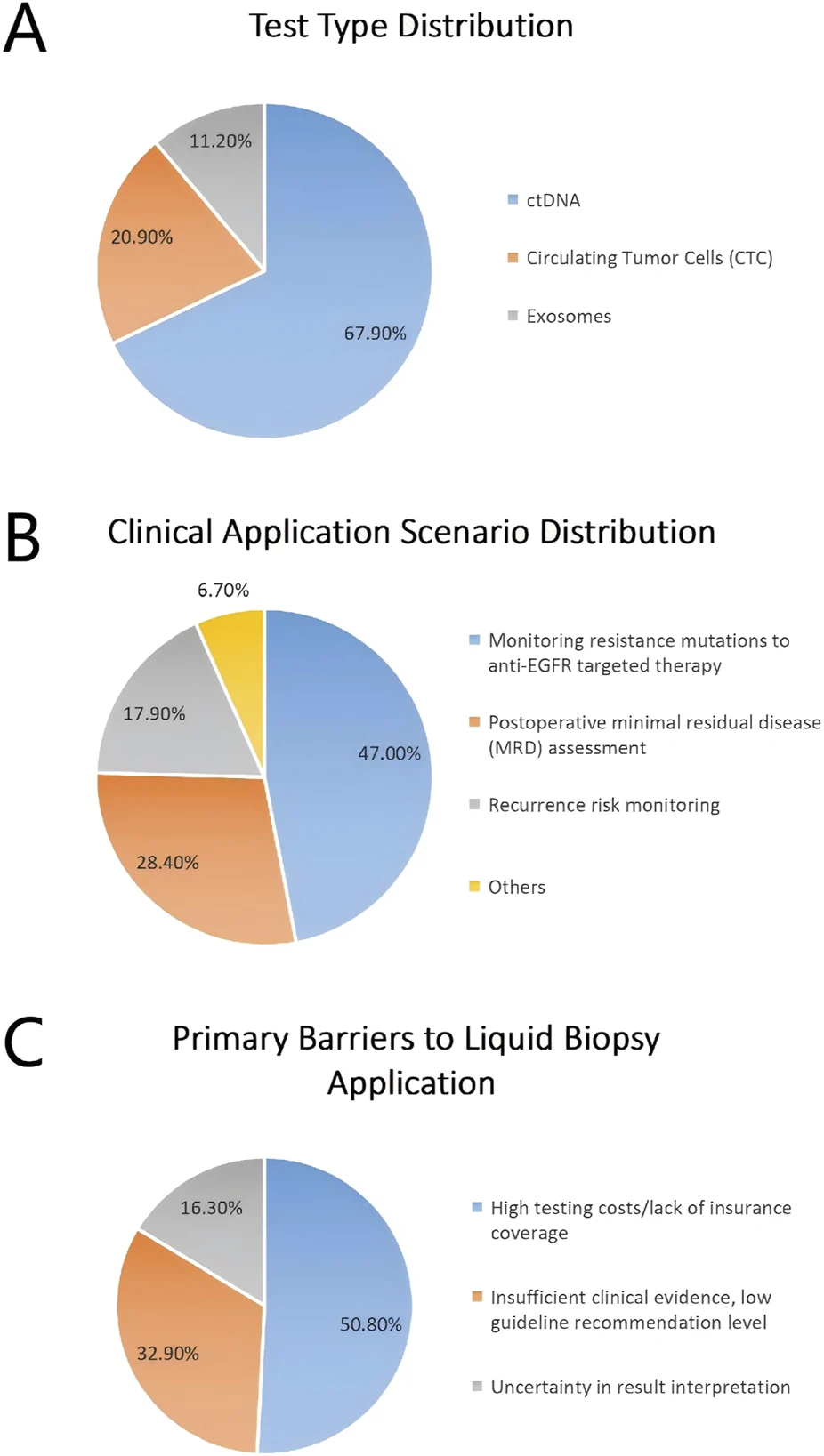
Analysis of Liquid Biopsy Utilization and Accessibility **(A)** Test type distribution. Among clinicians who had used liquid biopsy in practice (n = 134), circulating tumor DNA (ctDNA) was the most commonly used modality (67.9%), followed by circulating tumor cells (CTCs, 20.9%) and exosomes (11.2%). **(B)** Clinical application scenarios. Among clinicians with prior liquid biopsy experience (n = 134), the most common application was monitoring resistance mutations to anti-EGFR targeted therapy (47.0%), followed by postoperative minimal residual disease (MRD) assessment (28.4%) and recurrence risk monitoring (17.9%). Other applications accounted for 6.7%. **(C)** Primary barriers to liquid biopsy adoption. Among all respondents (N = 246), the most frequently reported barrier was high testing cost or lack of insurance coverage (50.8%), followed by insufficient clinical evidence and low guideline recommendation level (32.9%), and uncertainty in result interpretation (16.3%).

Among all respondents, 54.5% (134/246) of physicians indicated they had ordered LB for CRC patients in their clinical practice. The distribution of test types is shown in Figure 3A. Among the 134 physicians who used LB, ctDNA testing was overwhelmingly dominant (67.9%), while CTCs (20.9%) and exosomes (11.2%) were used relatively less frequently. There was a clear preference in the distribution of clinical application scenarios (Figure 3B). The most common scenario was monitoring resistance mutations to anti-EGFR targeted therapy (47.0%). This was followed by postoperative minimal residual disease (MRD) assessment (28.4%) and recurrence risk monitoring (17.9%). Other applications, including translational therapy efficacy evaluation and clinical trial enrollment, accounted for relatively lower proportions.

### 3.3 Key barriers to LB adoption

Analysis of barriers among all 246 respondents revealed critical bottlenecks (Figure 3C). “High testing costs/lack of insurance coverage” was cited as the primary barrier by the vast majority of physicians (50.8%). This was followed by “insufficient clinical evidence and low guideline recommendation levels” (32.9%) and “uncertainty in result interpretation” (16.3%). Interestingly, when asked whether they would incorporate LB into routine practice if cost and reimbursement issues were resolved, a substantial 89.8% (221/246) of physicians expressed willingness to do so.

### 3.4 Subgroup analysis and logistic regression results

**FIGURE 4:**
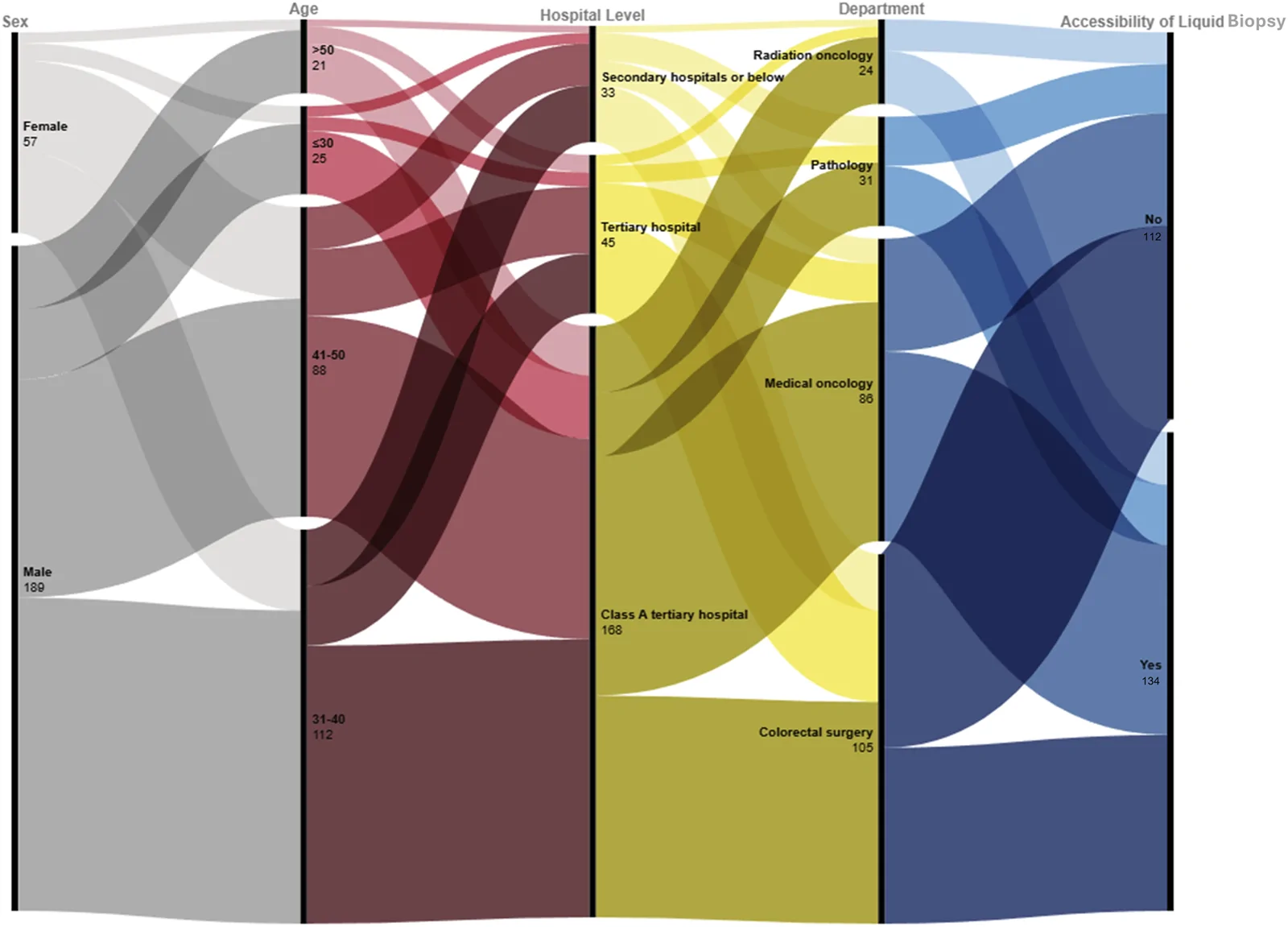
Sankey diagram illustrating the distribution of liquid biopsy accessibility across respondent demographic and institutional categories. The width of each flow is proportional to the number of respondents in that pathway. This diagram is purely descriptive and does not adjust for confounding between variables; flow widths reflect co-occurrence patterns in the sample and should not be interpreted as evidence of independent associations between any individual characteristic and LB accessibility.

Liquid biopsy (LB) adoption rates varied across physician subgroups with different characteristics. The Sankey diagram (Figure 4) provides a descriptive visualization of the distribution of LB accessibility across demographic and professional categories, showing how respondents from different subgroups are represented among those with and without LB access. As this diagram does not adjust for confounding, the observed flow patterns should not be interpreted as reflecting independent associations between individual variables and LB accessibility.

The utilization rate among physicians in Grade III Class A hospitals (65.5%) was significantly higher than in Grade III (non-Class A) hospitals (40.0%) and Grade II hospitals (24.2%) (P < 0.001). Similarly, institutions with annual CRC case volumes >100 (68.2% vs. 35.4%) and those with dedicated CRC treatment centers (68.1% vs. 35.3%) showed significantly higher usage rates. Univariate logistic regression analysis revealed that working in a Grade III Class A hospital (OR = 5.93, 95% CI 2.51–13.97), being designated as a CRC diagnosis and treatment center (OR = 2.88, 95% CI 1.71–4.88), and treating >100 cases annually (OR = 5.50, 95% CI 2.66–11.39) were the strongest predictors of LB accessibility (all P < 0.001). In the multivariable model, annual case volume >100 cases emerged as the sole independent predictor of LB accessibility after mutual adjustment (aOR = 4.11, 95% CI 1.57–10.77, P = 0.004). Notably, the association between CRC center designation and LB accessibility was substantially attenuated after adjustment for annual case volume (aOR = 1.33, 95% CI 0.65–2.71, P = 0.434), suggesting that this effect is largely mediated through higher case throughput rather than representing an independent institutional influence. Specialty category and professional title showed no significant association with LB accessibility in either univariate or multivariable analyses. The multivariable model demonstrated adequate fit (Hosmer-Lemeshow χ^2^ (8) = 5.88, P = 0.661; McFadden pseudo-R^2^ = 0.086). Full results are presented in Table 2.

**TABLE 2:** Univariate and multivariate logistic regression analysis of factors affecting the clinical application of liquid Biopsy (N = 246).

Subgroup	n (%)	Univariate analysis OR (95% CI)	P-value	Multivariate analysis OR (95% CI)	P-value
Hospital level^a^					
Grade II and below	33 (13.4)	**1.00 (ref)**	​	—	—
Grade III (non-Class A)	45 (18.3)	2.06 (0.75–5.68)	0.162	—	—
Grade III Class A	168 (68.3)	5.73 (2.41–13.61)	<0.001	—	—
CRC treatment center					
No	102 (41.5)	**1.00 (ref)**	​	**1.00 (ref)**	​
Yes	144 (58.5)	2.88 (1.70–4.87)	<0.001	1.33 (0.65–2.71)	0.434
Annual CRC case volume					
<50 cases	48 (19.5)	**1.00 (ref)**	​	**1.00 (ref)**	​
50–100 cases	58 (23.6)	2.68 (1.13–6.36)	0.03	2.16 (0.88–5.33)	0.094
>100 cases	140 (56.9)	5.49 (2.56–11.77)	<0.001	**4.11 (1.57–10.77)**	0.004
Discipline category					
Radiation oncology	24 (9.8)	**1.00 (ref)**	​	**1.00 (ref)**	​
Pathology/Other	31 (12.6)	0.73 (0.24–2.19)	0.569	0.98 (0.30–3.17)	0.976
Colorectal surgery	105 (42.7)	0.54 (0.22–1.34)	0.185	0.70 (0.27–1.84)	0.471
Medical oncology	86 (35.0)	1.01 (0.39–2.64)	0.979	1.10 (0.41–2.93)	0.854
Professional title					
Resident/Intern physician	22 (8.9)	**1.00 (ref)**	​	**1.00 (ref)**	​
Attending physician	120 (48.8)	0.62 (0.24–1.58)	0.317	0.75 (0.28–1.99)	0.559
Associate Chief physician	70 (28.5)	0.76 (0.28–2.06)	0.589	1.02 (0.36–2.91)	0.966
Chief physician	34 (13.8)	0.82 (0.27–2.50)	0.725	1.05 (0.33–3.36)	0.937

OR, odds ratio; aOR, adjusted odds ratio; CI, confidence interval. a The hospital level was excluded from the multivariate model because its variance inflation factor (VIF, 11.2) exceeded the multicollinearity threshold (VIF >10); univariate results are provided for reference only.

Bold values indicate statistically significant associations (P < 0.05).

## 4 Discussion

This study represents the first systematic cross-sectional survey of clinicians across different tiers of medical institutions in Zhejiang Province regarding the current status of LB application in CRC. Our core findings reveal that despite demonstrating significant potential as a cutting-edge non-invasive detection technology in CRC management, its clinical translation remains in its early stages, with a notable application gap persisting between institutions of varying healthcare resource levels. The findings clearly identify primary barriers to current adoption while reflecting clinicians' positive expectations for future implementation.

These results align with emerging literature on LB applications in oncology while highlighting regional disparities characteristic of middle-income countries like China. Globally, cost remains the foremost barrier to integrating LB into colorectal cancer management. Within our cohort, “high testing costs/lack of insurance coverage” was identified as the primary bottleneck by the vast majority of physicians, aligning closely with international observations. For instance, a comprehensive review by Samran et al. (Sheriff et al., 2025) on LB applications for cancer biomarkers noted that high testing fees and reimbursement gaps are prevalent barriers, often limiting patient access to advanced tests like ctDNA sequencing. Similarly, a U.S. perspective study on multi-cancer early detection (MCED) in blood found that despite over 90% awareness among healthcare practitioners, actual ordering rates remained below 10% (Schroll et al., 2024), primarily attributed to economic factors and insufficient evidence. Particularly under China’s current DRG/DIP payment reform for medical insurance, the adoption of high-cost technologies not yet covered by routine reimbursement faces more direct cost-control pressures within hospitals, which may be one of the underlying reasons for inter-hospital disparities.

Under these constraints, LB’s clinical application scenarios exhibit distinct preference distributions. In this study, LB’s most common clinical use—monitoring anti-EGFR resistance—accurately reflects its mature global application. LB demonstrates exceptional performance in detecting actionable mutations like RAS/BRAF in metastatic colorectal cancer. Multiple studies have confirmed that LB not only accurately detects RAS and BRAF mutations at baseline but also demonstrates high concordance with traditional tissue biopsies (Procaccio et al., 2021; Stintzing et al., 2025). Particularly in samples with tumor DNA content (TF) ≥1%, the positive concordance rates for RAS and BRAFV600E mutations reached 99% and 100%, respectively, with negative predictive values approaching 100% (Morris et al., 2025).

A notable finding is the relatively high application rate for MRD assessment and recurrence monitoring, which warrants careful contextualisation against the current evidence base. Landmark prospective studies have provided encouraging data: the DYNAMIC trial demonstrated that ctDNA-guided adjuvant chemotherapy decisions in stage II CRC reduced the proportion of patients receiving chemotherapy without compromising 2-year recurrence-free survival (Tie et al., 2022), and the GALAXY observational cohort within the CIRCULATE-Japan study reported that postoperative ctDNA positivity outperformed traditional clinicopathological features in predicting recurrence (Nakamura et al., 2024). These findings represent meaningful advances and help explain clinicians' enthusiasm for MRD monitoring in our survey. However, several important caveats temper their immediate clinical applicability. The DYNAMIC trial was powered for non-inferiority rather than superiority, and its sample size limits conclusions about overall survival benefit. The GALAXY cohort is observational, precluding causal inference about whether ctDNA-guided treatment modification improves outcomes compared to standard care. Crucially, neither study has yet demonstrated that acting on ctDNA-positive MRD results—rather than simply detecting them—translates into improved patient survival. The ESMO 2022 guidelines similarly acknowledge the prognostic value of ctDNA while explicitly recommending against routine clinical use outside of trial settings, citing insufficient evidence to guide treatment decisions (Pascual et al., 2022). Taken together, these considerations suggest that the enthusiasm for MRD monitoring documented in our survey may be running ahead of the evidence currently sufficient to justify routine practice adoption.

While physicians demonstrate enthusiasm for emerging MRD monitoring that aligns with international frontiers, their approach to other exploratory applications reveals notable caution. In stark contrast, the application rate of LB in translational therapy and clinical research settings was relatively low in this study. This may reflect clinicians' ongoing wait for more mature data and operational consensus in more complex treatment decisions, such as assessing the efficacy of neoadjuvant therapy and determining the optimal timing for surgery. This represents a gap compared to international frontiers, where ongoing multicenter trials demonstrate ctDNA’s predictive value across multiple tumor types. ctDNA clearance rates are now used as key secondary endpoints for assessing neoadjuvant therapy efficacy and predicting pathological complete response (pCR) (Wang et al., 2021; Magbanua et al., 2021; Ciriaco et al., 2022), while also aiding risk stratification, guiding “watchful waiting” strategies, and informing personalized treatment decisions (LaPelusa et al., 2024). This application disparity compounds inter-hospital disparities, as higher-tier centers have greater access to LB technology. Together, these factors paint a realistic picture of new technology adoption: even with compelling evidence, infrastructure, regulatory hurdles, and regional inequalities continue to impede equitable access to technology.

The interpretation challenges and evidence gap are frequently cited by respondents align with previous critiques. Although ctDNA analysis exhibits high sensitivity in advanced CRC, its application in early-stage disease and MRD detection demands exceptionally stringent technical requirements from testing platforms (Chen and Zhou, 2023; Semenkovich et al., 2023). These include analytical sensitivity, specificity, and the absence of standardized protocols—all contributing to clinicians' lack of confidence. By quantifying the weight of these barriers in a real-world Chinese cohort, this study offers a valuable Eastern perspective to the global discourse in this field.

The gap between clinician enthusiasm and guideline recommendations deserves direct engagement. The NCCN guidelines recognize ctDNA as an independent prognostic factor in stage II CRC, yet explicitly refrain from recommending its routine use to guide adjuvant treatment decisions outside of clinical trials (NCCN, 2026). This caution is substantive, not procedural. It reflects three unresolved concerns: first, the absence of randomized evidence demonstrating that ctDNA-guided treatment modifications improve overall survival; second, the analytical variability of ctDNA assays across platforms, which complicates cross-study comparisons and clinical standardization; and third, the risk of undertreating patients who test ctDNA-negative but harbor residual disease below the assay’s limit of detection. 54.5% of surveyed clinicians are already applying LB in practice indicates that real-world adoption is proceeding faster than the evidence base that guidelines are designed to reflect. This is not inherently problematic: early adoption in high-volume academic centers can generate the real-world data needed to accelerate guideline updates. However, it does underscore the importance of embedding LB use within structured data-collection frameworks and ensuring that patients are counselled about the current evidentiary limitations of ctDNA-guided decisions. Localized clinical evidence, operational consensus, and health economic evaluations serve as indispensable catalysts. The strong association with institutional factors in this study clearly indicates that cutting-edge technologies are concentrating in high-volume centers with abundant resources. This trend may objectively widen the “technology gap” between hospitals at different tiers. This trend of resource concentration toward high-volume centers creates tension with the original intent of China’s National Cancer Center in promoting regional medical centers and the tiered diagnosis and treatment system, reinforcing the dilemma of balancing ‘excellence’ and ‘equity’ when disseminating cutting-edge technologies. The multivariable analysis further refines this picture. Whereas CRC center designation appeared to be a significant predictor in univariate analysis, its effect was fully attenuated after adjustment for annual case volume, suggesting that the apparent benefit of center designation operates primarily through the higher case throughput that such institutions handle, rather than through any independent structural effect. This mediation pattern implies that case volume is the proximate driver of LB accessibility, a distinction with practical implications: policies aimed at expanding LB access may yield greater returns by concentrating CRC caseloads in high-volume centers than by expanding center designations alone. The non-significant findings for specialty category and professional title further reinforce this interpretation, pointing to a resource-availability framework in which access to LB is determined more by institutional capacity than by individual clinician characteristics. The absence of significant associations for specialty category and professional title warrants further comment. One might expect medical oncologists to report higher LB adoption than colorectal surgeons or radiation oncologists, given their closer engagement with systemic therapy decision-making. However, no such specialty gradient was observed, even after multivariable adjustment. Two explanations are plausible. First, subgroup sample sizes were limited—radiation oncologists (n = 24) served as the reference category, and these groups may have been underpowered to detect true specialty-level differences. Second, and perhaps more substantively, the finding is consistent with the resource-availability framework described above: if LB accessibility is primarily determined by institutional capacity rather than individual clinician characteristics, then who the clinician is matters less than where they work. This interpretation aligns with the broader pattern in our data, where institutional variables consistently dominated individual-level predictors.

From a clinical translation perspective, these findings chart a clear path forward. With 89.8% of physicians expressing willingness to incorporate LB into routine practice once reimbursement issues are resolved, this signals strong latent demand for broader adoption. However, this figure warrants cautious interpretation: hypothetical willingness expressed in survey settings is well-documented to overestimate actual future behavior, a phenomenon known as the intention-behavior gap. Even if reimbursement barriers were fully removed, real-world adoption would additionally require workflow integration, clinician training, and greater guideline clarity on when and how to act on LB results. Insurance coverage should therefore be understood as a necessary but not sufficient condition for driving adoption, and policy efforts should address these complementary implementation barriers in parallel. This mirrors experiences in some European countries where ctDNA inclusion in state-funded clinical research and practice programs significantly accelerated technological translation. Furthermore, the Sankey diagram offers a descriptive overview of how LB accessibility is distributed across subgroups, providing a useful visual reference for identifying which clinician populations remain underserved. Priority should be given to standardized training for physicians in mid-tier hospitals and non-oncology specialists to bridge the gap between knowledge and practice. These insights, grounded in China’s real-world data, are not only crucial for optimizing precision diagnosis and treatment of CRC but also provide a highly valuable blueprint for the standardized application of LB technology in early screening for other cancers and across cancer types.

## 5 Limitations

Limitations of this study include: recruitment through provincial academic societies, specialty alliances, and academic conferences introduces potential selection bias. Clinicians who participate in academic networks are likely more engaged with emerging technologies and thus more likely to have already adopted LB. This systematic over-representation of academically active clinicians means that the 54.5% utilization rate reported here may overestimate the true adoption rate among all Zhejiang CRC clinicians, particularly those practicing in community hospitals, rural settings, or primary care institutions not reached by these academic channels. A more substantive limitation is the absence of platform-level differentiation. Clinician perceptions of evidence quality, cost acceptability, and result interpretability differ meaningfully between NGS-based and ddPCR-based approaches, as these platforms have distinct analytical sensitivities, turnaround times, and cost profiles. By aggregating all ctDNA modalities under a single category, our data may obscure meaningful heterogeneity in both adoption patterns and perceived barriers. This limitation may materially affect the interpretation of findings related to barriers and clinical scenarios. Future survey instruments should incorporate platform-specific questions to capture this granularity. The achieved sample size (N = 246) fell short of the pre-specified requirement of 385, increasing the margin of error from the intended ±5.0% to approximately ±6.3% at the 95% confidence level. This shortfall may have reduced statistical power for subgroup comparisons—particularly for smaller reference categories such as radiation oncologists (n = 24) and residents (n = 22)—and likely contributed to the wide confidence intervals observed for several odds ratio estimates in Table 2. Non-significant associations should therefore be interpreted with caution, as they may reflect insufficient power rather than true absence of effect. Prospective multicenter trials should be conducted to validate the actual impact of LB -guided treatment decisions on clinical outcomes. Additionally, cost-effectiveness analyses tailored to China’s healthcare economic context should be performed to provide more direct evidence for health insurance policy development.

## 6 Conclusion

In summary, this regional survey provides implementation-level evidence on clinician-reported liquid biopsy adoption in CRC care in Zhejiang Province, revealing significant potential yet insufficient clinical translation, alongside pronounced regional disparities. Cost, evidence, and accessibility constitute the primary barriers to current adoption. This study both aligns with and expands upon the global literature, advocating for policy reforms, localized clinical research, and targeted educational outreach to fully unlock LB's potential in improving patient outcomes.

## Data Availability

The datasets presented in this article are not readily available because the data that support the findings of this study are not publicly available but can be obtained from the corresponding author upon reasonable request. Requests to access the datasets should be directed to YC, iloveurandy163@hotmail.com.
