# Correction: Unequal access to liquid biopsy in colorectal cancer care: a cross-sectional survey of clinicians across hospital tiers in Zhejiang, China

**DOI:** 10.3389/fcell.2026.2003239

**Published:** 2026-10-07

**Authors:** Sheng Li, Zhonglei Shen, Youchang Sun, Yitong Hu, Yisheng Cao

**Affiliations:** 1 Department of Anorectal Surgery, Ningbo No. 2 Hospital, Wenzhou Medical University, Ningbo, Zhejiang, China; 2 Health Science Center, Ningbo University, Ningbo, Zhejiang, China

**Keywords:** accessibility, circulating tumor DNA, clinical adoption, colorectal cancer, liquid biopsy

Affiliation 1 “Department of Anorectal Surgery, Ningbo No.2 Hospital, Wenzhou Medical University, Ningbo, Zhejiang, China” was erroneously given as “Department of Anorectal Surgery, Wenzhou Medical University, Ningbo, Zhejiang, China”.

The funder “the Science and Technology Innovation Yongjiang 2035 Key Research and Development Project of Ningbo, Project ID: 2024Z229” was erroneously omitted. The correct funding statement reads:

The author(s) declared that financial support was received for this work and/or its publication. This research is supported by HwaMei Research Foundation of Ningbo No. 2 Hospital, Grant No: 2024HMKYA57, Zhejiang Provincial Medical and Health Research Project, Project ID: 2022KY1121 and the Science and Technology Innovation Yongjiang 2035 Key Research and Development Project of Ningbo, Project ID: 2024Z229.

The original version of this article has been updated.

