# Supplementary material for: Unequal access to liquid biopsy in colorectal cancer care: a cross-sectional survey of clinicians across hospital tiers in Zhejiang, China
Source: Front Cell Dev Biol. 2026 Aug 6;14:1870567. doi: 10.3389/fcell.2026.1870567 (PMC13490070; doi:10.3389/fcell.2026.1870567)
Supplement: Supplementary file 1 [file DataSheet1.docx]

Dear colleague,

We are conducting a survey to understand the current accessibility, utilization patterns, perceived barriers, and future perspectives regarding liquid biopsy in the clinical management of colorectal cancer (CRC) in Zhejiang Province, China.

This questionnaire is intended for healthcare professionals involved in CRC diagnosis, treatment, pathology, or related research. Participation is voluntary and anonymous. No personally identifiable information will be collected. The survey results will be used for academic research purposes only.

By proceeding to the questionnaire, you confirm that you have read the above information and agree to participate in this study.

List of questions.

Questionnaire (English Version)

1. Age

Please select your age group:

□ ≤30 years

□ 31–40 years

□ 41–50 years

□ >50 years

2. Gender

□ Male

□ Female

3. Professional title

□ Resident physician / Intern

□ Attending physician

□ Associate chief physician

□ Chief physician

□ Other: __________

4. Primary specialty/discipline

□ Colorectal surgery

□ Medical oncology

□ Radiation oncology

□ Pathology

□ Gastroenterology

□ General surgery

□ Laboratory medicine / molecular diagnostics

□ Research staff

□ Other: __________

5. Type/level of hospital where you currently work

□ Grade III Class A hospital

□ Grade III hospital (non-Class A)

□ Grade II hospital or below

□ Other: __________

6. Does your institution have a designated colorectal cancer diagnosis and treatment center?

□ Yes

□ No

□ Not sure

7. Approximate annual number of CRC cases managed at your institution

□ <50 cases

□ 50–100 cases

□ >100 cases

□ Not sure

8. How many years have you been involved in CRC-related clinical practice or research?

□ <1 year

□ 1–3 years

□ 4–10 years

□ >10 years

9. Geographic location of your institution

City/Region: __________

Module 2. Accessibility and Utilization of Liquid Biopsy in CRC

10. Are you familiar with the concept of liquid biopsy in colorectal cancer?

□ Very familiar

□ Somewhat familiar

□ Heard of it, but not familiar with details

□ Not familiar

11. Has liquid biopsy ever been used or ordered in your clinical practice for CRC patients?

□ Yes

□ No

12. Does your institution have access to liquid biopsy testing for CRC patients?

□ Yes, in-house testing is available

□ Yes, but mainly through external laboratories

□ No, currently unavailable

□ Not sure

13. If liquid biopsy is available in your institution, how is it usually performed?

□ In-house laboratory testing

□ Sent to an external laboratory/company

□ Both in-house and external testing

□ Not applicable

□ Not sure

14. Which types of liquid biopsy are available or used in your practice?

(Multiple answers allowed)

□ ctDNA

□ Circulating tumor cells (CTCs)

□ Exosomes

□ DNA methylation markers

□ Fragmentomics

□ Other: __________

□ Not sure

15. What is the typical turnaround time for liquid biopsy reports in your setting?

□ <3 days

□ 3–7 days

□ 8–14 days

□ >14 days

□ Not sure

□ Not applicable

16. Who usually initiates or recommends liquid biopsy in CRC patients in your institution?

□ Surgeon

□ Medical oncologist

□ Pathologist

□ Multidisciplinary team (MDT)

□ Laboratory/molecular tumor board

□ Other: __________

□ Not sure

For respondents who answered “Yes” to Question 11

17. Which liquid biopsy modality have you used most frequently in CRC practice?

□ ctDNA

□ CTCs

□ Exosomes

□ Methylation markers

□ Other: __________

18. In which clinical scenarios have you used liquid biopsy for CRC?

(Multiple answers allowed)

□ Postoperative minimal residual disease (MRD) assessment

□ Recurrence surveillance/follow-up

□ Monitoring resistance mutations during anti-EGFR therapy

□ Re-challenge decision-making for targeted therapy

□ Treatment response evaluation during systemic therapy

□ Evaluation of neoadjuvant/conversion therapy efficacy

□ Prognostic assessment / risk stratification

□ Patient selection for clinical trials

□ Early screening / early detection

□ Other: __________

19. Which single scenario do you consider the most clinically useful at present?

□ Postoperative MRD assessment

□ Recurrence surveillance/follow-up

□ Monitoring resistance to anti-EGFR therapy

□ Re-challenge decision-making

□ Prognostic assessment

□ Clinical trial enrollment

□ Other: __________

20. In your experience, how clinically useful is liquid biopsy for CRC overall?

□ Very useful

□ Useful

□ Neutral

□ Of limited usefulness

□ Not useful

21. How confident are you in interpreting liquid biopsy results in CRC?

□ Very confident

□ Fairly confident

□ Neutral

□ Slightly unconfident

□ Not confident at all

22. How often do liquid biopsy results influence your actual clinical decisions?

□ Very often

□ Often

□ Sometimes

□ Rarely

□ Never

Module 3. Perceived Barriers, Attitudes, and Future Perspectives

23. What do you consider the main barrier to wider clinical adoption of liquid biopsy in CRC?

(Select one)

□ High cost / lack of insurance reimbursement

□ Insufficient clinical evidence

□ Limited accessibility / testing availability

□ Difficulty interpreting results

□ Inadequate laboratory quality control / standardization

□ Poor patient acceptance

□ Long turnaround time

□ Lack of institutional support

□ Other: __________

24. Which of the following barriers have limited the clinical use of liquid biopsy in your setting?

(Multiple answers allowed)

□ High cost

□ Lack of insurance reimbursement

□ Insufficient clinical evidence

□ Low guideline recommendation level

□ Difficulty interpreting results

□ Limited access to testing platforms

□ Inadequate laboratory quality control

□ Uncertainty regarding test accuracy

□ Long turnaround time

□ Poor patient acceptance

□ Lack of clinician training

□ Lack of institutional support

□ Other: __________

25. In your opinion, which factor is most urgently needed to promote wider use of liquid biopsy in CRC?

□ Insurance reimbursement / reduced cost

□ More high-quality clinical evidence

□ Clearer guideline recommendations

□ Better clinician education and training

□ Improved laboratory standardization/quality control

□ Improved access to testing platforms

□ Faster report turnaround time

□ Other: __________

26. To what extent do you agree with the following statement?

“Current evidence supporting liquid biopsy in CRC is sufficient for broader routine clinical use.”

□ Strongly agree

□ Agree

□ Neutral

□ Disagree

□ Strongly disagree

27. To what extent do you agree with the following statement?

“I am confident that liquid biopsy results can be reliably integrated into CRC clinical decision-making.”

□ Strongly agree

□ Agree

□ Neutral

□ Disagree

□ Strongly disagree

28. If liquid biopsy became more accessible and/or covered by medical insurance, would you be willing to incorporate it into routine CRC clinical practice?

□ Yes

□ No

□ Not sure

29. In which CRC settings do you think liquid biopsy is most likely to become routine in the future?

(Multiple answers allowed)

□ MRD detection after surgery

□ Recurrence monitoring during follow-up

□ Resistance monitoring in metastatic CRC

□ Re-challenge strategy guidance

□ Early screening / early diagnosis

□ Treatment response assessment

□ Clinical trial stratification

□ Other: __________

30. What kind of support would most help you use liquid biopsy more effectively?

(Multiple answers allowed)

□ Institutional reimbursement support

□ National/provincial insurance coverage

□ More robust clinical evidence

□ Consensus guidelines / standardized pathways

□ Interpretation support from molecular tumor boards

□ More training courses/workshops

□ Better access to accredited laboratories

□ Faster turnaround time

□ Other: __________

31. Additional comments or suggestions regarding the use of liquid biopsy in CRC clinical practice:

Open-ended response:
